# *γ*-Aminobutyric Acid (GABA)-enriched Hemp Milk by Solid-state Co-fermentation and Germination Bioprocesses

**DOI:** 10.1007/s11130-024-01187-6

**Published:** 2024-05-16

**Authors:** Gulsah Karabulut, Boris V. Nemzer, Hao Feng

**Affiliations:** 1 Department of Food Engineering, Sakarya University, Sakarya, 54187 Turkey; 2 VDF FutureCeuticals, Inc, Momence, IL 60954 USA; 3 Department of Family and Consumer Sciences, North Carolina A&T State University, Greensboro, NC 27411 USA

**Keywords:** GABA, Hemp milk, Probiotic strains, Solid-state fermentation, Germination

## Abstract

This study introduces the concept of developing a functional hemp drink enriched with *γ*-Aminobutyric acid (GABA) to enhance its nutritional value and functional properties utilizing Solid-State (SSF) co-Fermentation by *Lactobacillus casei* and *Bacillus subtilis* and germination bioprocesses. Bioprocesses may offer an alternative solution to challenges in hemp milk, such as product instability and the use of additives. Notably, the hemp milk produced through the germination for three days or co-fermentation processes yielded the highest GABA content of 79.84 and 102.45 mg/100 mL, respectively, compared to the untreated milk. These bioactive milk samples exhibited higher zeta potential and soluble protein content and also reduced solid particle sedimentation and droplet sizes (*D*_*4,3*_ and *D*_*3,2*_) compared to the untreated milk. Furthermore, the peptide, total phenolic content, and antioxidant activity of the produced GABA-enriched kinds of milk surpassed those of the untreated milk. Overall, the SSF and germination processes present a promising alternative for producing stable milk analogs with enhanced health-boosting properties.

**Supplementary Information:**

The online version contains supplementary material available at 10.1007/s11130-024-01187-6.

## Introduction

Nutraceutical and functional components, such as *γ*-aminobutyric acid (GABA), are experiencing rapid growth in the food industry. This growth is especially prevalent in the beverage sector, where bioactive compounds are incorporated into products [1]. This trend has led to the expansion of non-dairy milk substitutes like soy, hemp, almond, and coconut milk in the global milk alternatives market. The popularity of non-dairy milk substitutes, which is expected to reach US$123.1 billion by 2030, can be attributed to various factors, including ethical, environmental, and animal welfare concerns [2]. γ-aminobutyric acid is a naturally occurring compound that can positively impact mood, cognitive function, relaxation, and sleep quality [3, 4]. Natural GABA can be produced through microbial fermentation by probiotic strains and germination, offering a safer and more environmentally friendly alternative to synthetic GABA production [5]. While many studies have used single strains of lactic acid bacteria (LAB) to produce GABA, there are limited reports on co-culturing for GABA production, indicating the potential of this approach [6, 7].

Hemp seeds, sourced from *Cannabis sativa* L., are a rich source of essential nutrients (25% protein and 30% oil), amino acids, and beneficial fatty acids, making them a promising alternative to traditional dairy, soy, and nut-based milk substitutes [8]. Recent research has revealed various health benefits of hemp seed consumption, including immune system regulation, neuroprotection, and cardiovascular benefits [9]. Hemp seeds also contain compounds like cannabidiolic acid (CBDA) and arginine, which may protect against chronic diseases [10]. Additionally, the high glutamate content in hemp seeds makes them suitable for increasing GABA levels through fermentation and germination.

**Fig. 1:**
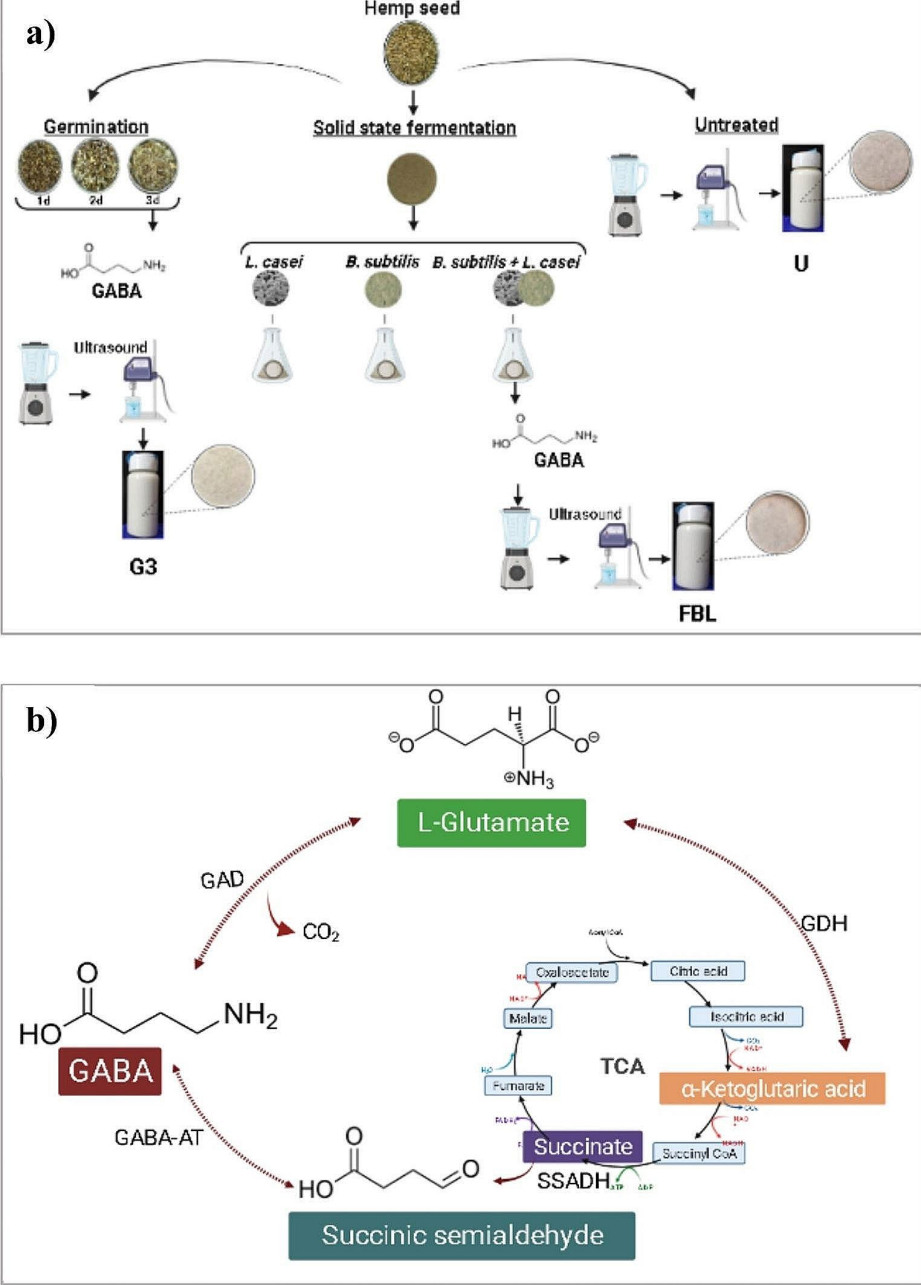
**(a)** The schematic diagram of hemp milk production from germinated, solid-state fermented, and untreated seeds and **(b)** summary of the GABA biosynthesis pathway. U: untreated; G3: germinated for 3 days; FBL: solid state fermented by co-cultures of. *L. casei* and *B. subtilis*, GABA: *γ*-aminobutyric acid; GAD: glutamic acid decarboxylase; GDH: glutamic acid dehydrogenase; TCA: tricarboxylic acid; SSADH: succinate-semialdehyde decarboxylase; GABA-AT: GABA transaminase

Hemp milk is a popular and convenient plant-based beverage with a nutrient profile similar to cow’s milk but without cholesterol, gluten, or lactose. Despite its benefits, hemp milk faces stability challenges related to its oil-in-water emulsion [11]. Bioprocesses, including solid-state fermentation and germination, have emerged as promising alternatives to synthetic stabilizers, offering cost-effectiveness, environmental friendliness, safety, sustainability, and ease of implementation [12, 13]. Hemp seeds contain prebiotic ingredients such as oligosaccharides, fibers, polyphenols, and isoflavones. Solid-state fermentation aids in the partial breakdown of organic substances, such as complex protein and protein-phenol structures, yielding smaller, soluble forms [11]. The primary objectives of this study were to compare the impact of fermentation utilizing probiotic strains, including *Lactobacillus casei* and *Bacillus subtilis*, and their co-cultures and germination on GABA and antioxidant production, evaluate the effect of solid-state co-fermentation, and investigate the influence of bioprocesses on the physical properties of hemp milk (Fig. 1a). This is the first attempt to examine the GABA content in hemp seeds and their fermented and germinated derivates.

## Materials and Methods

The section on materials and methods is shown in Supplementary File S1.

## Results and Discussion

### GABA Content

In solid-state fermentation using *L. casei*, *B. subtilis*, and their co-cultures on hemp seeds, higher GABA contents were observed compared to germinated seeds for up to 3 days (Table 1- see in S2). The FBL seeds that co-fermented with *L. casei* and *B. subtilis* displayed the highest GABA content at 175.98 mg/100 g dry weight (dw), surpassing untreated seeds by 10.28 times. FB seeds had a GABA content of 152.38 mg/100 g dw, and FL seeds had a GABA content of 128.51 mg/100 g dw, representing 8.90 and 7.51 times higher GABA levels than untreated seeds, respectively. Through solid-state fermentation using single and co-cultures of *L. casei* and *B. subtilis*, as well as germination for up to 3 days, the GABA content in hemp milk formulations ranged from 14.08 to 102.45 mg/100 mL (Table 1- see in S2). As seen in Fig. 1b, GABA synthesis from L-glutamate is facilitated by the GAD enzyme, which various microorganisms, including LAB, yeasts, and fungi, can produce. The synthesis of GABA occurs via the enzymatic decarboxylation of L-glutamate facilitated by glutamic acid decarboxylase (GAD). The GAD is formed from α-ketoglutaric acid in the tricarboxylic acid (TCA) cycle through the action of glutamic acid dehydrogenase (GDH) [14–16]. The amount of glutamic acid in the raw products influences the GABA content in sprouts. The increased GABA content observed during seed germination can be attributed to the breakdown of seed-storage compounds and the synthesis of structural proteins and other cellular components necessary for plant growth. In higher plants, GABA biosynthesis primarily occurs through the GABA shunt pathway, with the polyamine degradation pathway playing a secondary role. The GABA shunt pathway involves the decarboxylation of glutamate to produce GABA, catalyzed by GAD. GABA is subsequently converted to succinate semialdehyde by GABA transaminase (GABA-AT). Succinate semialdehyde is further oxidized by succinate semialdehyde decarboxylase (SSADH) to form succinic acid, which enters the tricarboxylic acid cycle (TCA cycle) to provide a carbon skeleton for ATP synthesis in plants [20]. The synthesis of GABA from L-glutamate in metabolism is facilitated by the GAD enzyme, which can be produced by various microorganisms such as LAB, yeasts, and fungi [34]. Previous studies have reported GABA production levels of 50.4 mg/kg for *L. casei,* and 15.40 mg/mL for *B. subtilis* [17, 18]. In the co-culture fermentation of *L. casei* and *B. subtilis*, there appears to be a synergistic enhancement in GABA bioconversion compared to their individual fermentations. Initially, *B. subtilis* dominated the fermentation of hemp seeds. Still, as conditions became acidic and anaerobic, *L. casei* became the predominant microorganism, a condition known to increase GABA production in species like *L. brevis* and *L. plantarum*. Thus, the anaerobic conditions and a limited carbon source likely facilitated L-glutamate conversion to GABA [19]. Recent studies on co-culture fermentation and GABA production support these findings, suggesting that co-culture solid-state fermentation holds promise for GABA generation [20–22].

### Zeta Potential and Droplet Sizes

**Fig. 2:**
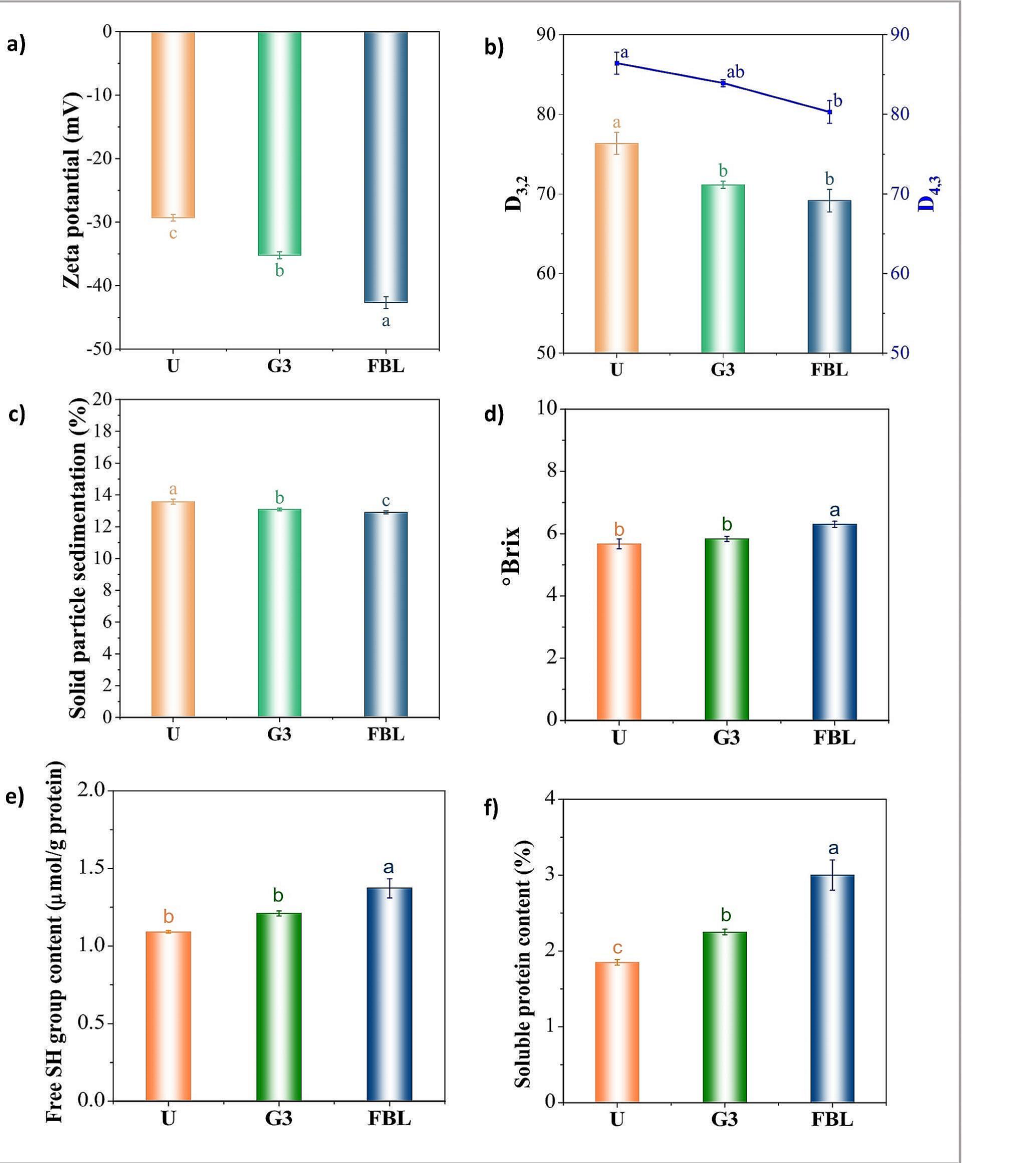
**(a)** Zeta potential, **(b)** droplet sizes (*D3,2*, *D4,3*), **(c)** solid particle sedimentation, **(d)** brix, **(e)** free -SH group content (µmol/g protein), and **(f)** soluble protein content (%) of hemp milk samples. U: untreated; G3: germinated for 3 days; FBL: solid state fermented by co-cultures of. *L. casei* and *B. subtilis*

Figure 2a and b show the zeta potential and droplet sizes of hemp milk samples. The FBL milk exhibited the highest zeta potential values among the milk samples, negatively increasing to - 42.68 mV. Compared to the untreated milk sample (-29.31 mV), the G3 (-35.2 mV) and FBL milk samples had zeta potentials that were 17 and 31% higher, respectively. Zeta potential values exceeding ±25 mV commonly indicate improved colloidal stability. High values suggest electrostatic solid repulsion between particles, effectively preventing their aggregation and sedimentation within the colloidal suspension [23]. Zeta potential measurements aligned with particle size distribution data, showing that both *D3,2* and *D4,3* values in the G3 and FBL samples were lower than in the untreated hemp milk. The FBL sample had the lowest *D3,2* and *D4,3* values, with a reduction of 9.4% and 7.1% compared to the untreated sample. Hemp milk’s high-fat macromolecules and globular edestin protein with high crystallinity contribute to low solubility and protein-protein aggregation [24]. Solid-state fermentation and germination processes enzymatically break down these macromolecules, forming more minor, more soluble subunits [25].

### Solid Particle Sedimentation and ºBrix

Figure 2c demonstrates a slight reduction in solid particle sedimentation in the FBL and G3 milk samples compared to the untreated milk, leading to improved physical stability. Bioprocessing methods effectively mitigate the tendency of macroaggregates to coalesce, resulting in a reduction in particle size. This leads to the formation of smaller protein aggregates with surface-active properties, enhancing the development of a more efficient film layer around oil droplets and, consequently, bolstering emulsion stability [26]. The noticeable increase in the ºBrix measurement of the FBL sample, compared to both the untreated and G3 milk samples, may be attributed to improved solubility (Fig. 2d). The presence of exopolysaccharides generated through bacterial fermentation and carbohydrates decomposed during the germination process may contribute to increased viscosity in hemp milk, resulting to a more stable product. Consequently, bioprocessing methods are a viable and cost-effective alternative to synthetic stabilizers.

### Free Sulfhydryl (-SH) Group Content

Thiol, which contains free -SH functional groups, is an essential antioxidant in preventing oxidative stress-induced damage and protects cells against oxidative stress [19]. As illustrated in Fig. 2e, the FBL milk samples exhibited a higher concentration of free -SH groups than the untreated and G3 samples. The observed phenomenon can be attributed to the activation of endogenous proteases during the fermentation and germination process. These proteases, secreted by microorganisms, enzymatically cleave the peptide bonds within the protein, resulting in the unfolding of the polypeptide chain and subsequent disruption of the disulfide bonds associated with the active sulfhydryl groups situated within the protein molecule [27]. These processes lead to an increase in the abundance of free sulfhydryl groups.

### Soluble Protein Content

Figure 2f shows the influence of the SSF and germination bioprocesses on the protein solubility of hemp milk samples. As depicted, the FBL and G3 milk samples exhibited higher protein solubility of 3.00 and 2.25 g/100 mL, respectively, a 38 and 25% increase compared to the untreated milk sample. Biotechnological processes reduced particle size, as seen in Fig. 2b, leading to increased surface charge on proteins, including hydrophilic regions. This enhanced surface charge can promote converting insoluble protein forms into soluble forms through hydrolysis and partial denaturation [28]. Amino acid transformation by *de novo* synthesis and increased hydrophilic amino acid content [27], such as lysine, during bioprocesses, can improve the water affinity of protein isolates, leading to increased solubility.

### Peptide Content

**Fig. 3:**
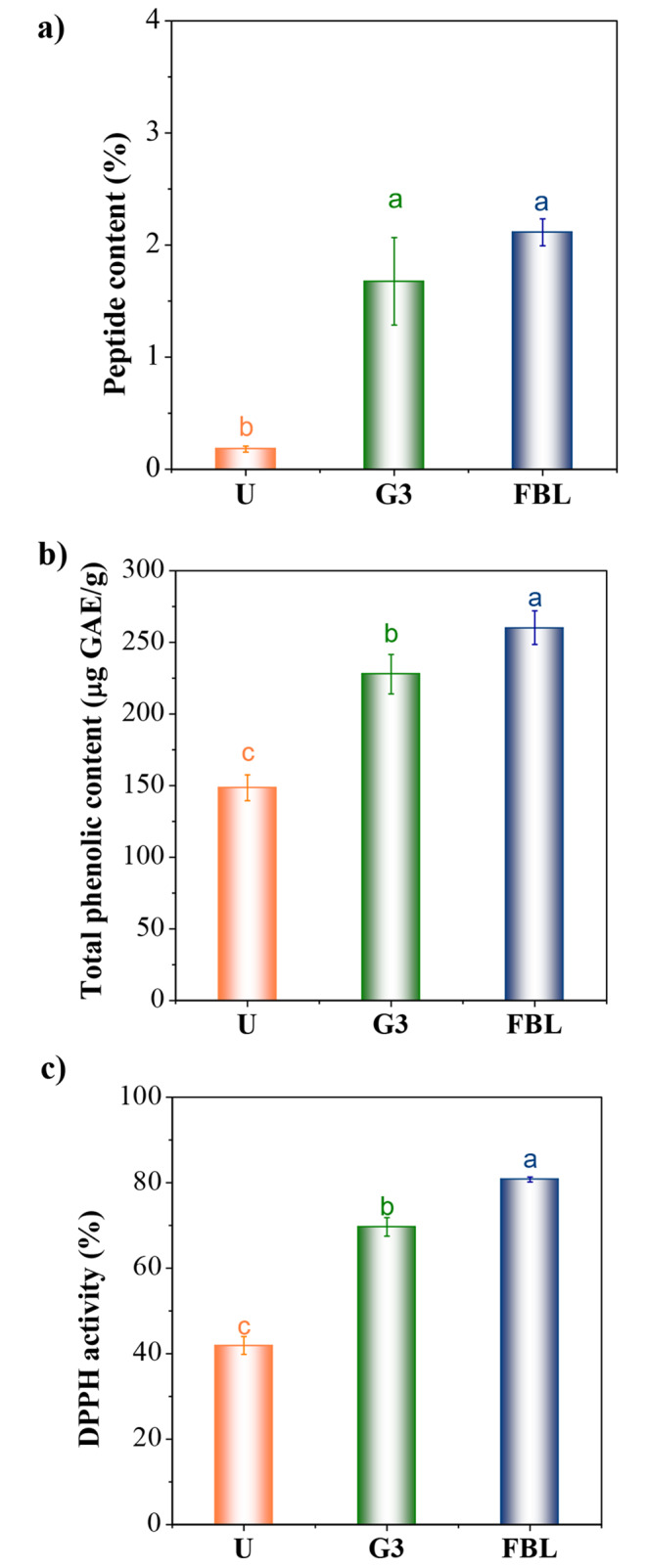
**(a)** Peptide content (%), **(b)** DPPH activity (%), and **(c)** total phenolic content (µg GAE/g) of hemp milk samples. U: untreated; G3: germinated for 3 days; FBL: solid state fermented by co-cultures of. *L. casei* and *B. subtilis*

The peptide content of hemp milk samples is depicted in Fig. 3a. The bioprocesses of the SSF and germination resulted in increased peptide content within the milk samples. Specifically, the G3 and FBL milk samples exhibited peptide content that was 9.3 and 11.7 times higher, respectively, compared to the untreated milk. Germination is a physiological process that stimulates the disintegration of plant cell structures and the hydrolysis of proteins through activating enzymes, including hydrolases, proteases, and peptidases [27]. This intricate process entails the mobilization of storage proteins via endopeptidases and carboxypeptidases, leading to the breakdown of proteins into amino acids to serve as an energy source [26]. Consequently, the proteins undergo hydrolysis, forming low molecular weight amino acids and peptides, thereby augmenting protein solubility. Likewise, bacterial proteolytic enzymes from co-cultures of L. casei and *B. subtilis* during solid-state fermentation facilitate the degradation of large protein aggregates into peptides. Both germination and fermentation processes involve the activation of hydrolytic enzymes to facilitate protein degradation and release smaller peptide constituents [29, 30]. The proteolytic activity exhibited by *Lactobacillus* spp. enables the enzymatic degradation of proteins into specific bioactive peptides that display inhibitory properties against α-amylase and α-glucosidase [31]. Numerous studies have reported on the antioxidant and anti-hypertensive attributes of hemp seed peptides and protein hydrolysate [32]. Therefore, the field of bioprocess engineering is experiencing a surge in interest regarding biologically active peptides derived from proteins.

### Total Phenolic Content and Antioxidant Activity

The total phenolic content of hemp milk samples was measured, and the results showed that the FBL sample exhibited the highest phenolic content, with an impressive increase of over 75% compared to the untreated milk sample (Fig. 3b and c). The G3 milk sample also displayed a substantial increase, with a 53% rise in phenolic content. Additionally, the DPPH reducing activity (%) significantly increased in the FBL and G3 milk samples, with enhancements of 92.97 and 66.40%, respectively, compared to the untreated milk samples. Several factors contribute to the increase in total phenolic content and antioxidant activities during germination and fermentation processes. Bioprocessing techniques like germination and fermentation break down protein-phenolic complexes, converting bound phenolic compounds into free forms through proteolytic activities [33, 34]. Fermentation, driven by *β*-glucosidase enzymes, further hydrolyzes phenolic-glucosides and phenolic-protein conjugates, increasing free phenolic concentrations [27, 35]. These processes generate essential intermediate compounds for phenolic biosynthesis [36]. Microbial enzymatic activity and metabolic processes can create new phenolic compounds facilitated by changes in pH and distribution of amino acids [25, 36]. Amino acids with antioxidant properties, such as histidine, tyrosine, methionine, and cysteine, correlate positively with increased antioxidant activity. Various enzymatic processes catalyze proteolysis, leading to higher levels of potentially bioactive peptides and active polyphenolic compounds, enhancing antioxidant activity [8].

## Conclusions

This study utilized the solid-state fermentation and germination processes to enhance the nutritional value and functionality of hemp milk, resulting in a GABA-enriched product. Despite the challenges related to hemp milk stability and additives, SSF and germination proved to be viable alternatives. The G3 and FBL milk samples exhibited substantial increases in GABA levels, surpassing untreated seeds by 6.6 and 10.8 times, respectively, potentially offering health benefits. These improved samples also showed higher zeta potential and lower droplet sizes, indicating increased stability. Both bioprocesses contributed to higher phenolic, antioxidant, and peptide contents, enhancing the milk’s potential as a health-promoting beverage. Overall, the study demonstrated the potential of GABA-enriched hemp milk as a unique and healthful option.

### Electronic Supplementary Material

Below is the link to the electronic supplementary material.

Supplementary Material 1

Supplementary Material 2

## Data Availability

No datasets were generated or analysed during the current study.
