# Supplementary material for: γ-Aminobutyric Acid (GABA)-enriched Hemp Milk by Solid-state Co-fermentation and Germination Bioprocesses
Source: Plant Foods Hum Nutr. 2024 May 16;79(2):322–9. doi: 10.1007/s11130-024-01187-6 (PMC11178579; doi:10.1007/s11130-024-01187-6)
Supplement: Supplementary file 1 — Supplementary Material 1 [file 11130_2024_1187_MOESM1_ESM.docx]

**γ-Aminobutyric acid (GABA)-enriched hemp milk by solid-state co-fermentation and germination bioprocesses**

Gulsah Karabulut^a^, Boris V. Nemzer^b^, Hao Feng^c,^*

^a^Department of Food Engineering, Sakarya University, 54187, Sakarya / Turkey ^b^VDF FutureCeuticals, Inc., Momence, IL 60954, USA ^c^Department of Family and Consumer Sciences, North Carolina A&T State University, 27411, Greensboro, NC / USA

*Corresponding author: Hao Feng [hfeng@ncat.edu](mailto:hfeng@ncat.edu)

Supplementary File 1

**Materials and Methods**

**Materials**

The certified industrial hemp seeds (USO 31 variety) harvested in 2021 from the Urbana-Champaign Area (USA) were kindly donated by the Department of Crop Sciences at the University of Illinois at Urbana-Champaign (USA). The hemp seed used in this study had 22.8% protein and 36.7% fat content. The probiotic bacteria *L. casei* (ATCC 393) or *B. subtilis* (ATCC 23857) were obtained from the culture collection of Dr. Miller at the University of Illinois at Urbana-Champaign (USA) in the Department of Food Science and Human Nutrition. All chemicals were purchased from Merck (Darmstadt, Germany) and Sigma (Steinheim, Germany) at an analytical or higher grade.

**Germination**

In this study, hemp seeds were washed with distilled water and then sterilized with a 0.07% NaClO solution for 30 minutes. The seeds were thoroughly rinsed to remove any remaining NaClO solution. Next, the seeds were soaked in distilled water for 8 hours at 23 °C. The soaked seeds were placed in a dark environment at 23 °C for germination, which occurred throughout 1, 2, and 3 days by placing the seeds between two layers of wet qualitative filter paper. After germination, the hemp seeds were dried at 45 °C in an oven and ground [1]. Finally, the germinated hemp seeds were immediately used to produce fresh hemp milk samples.

**Solid-state fermentation**

The solid-state fermentations (SSF) were conducted following a modified method described by [2]. The probiotic bacteria, *L. casei* (ATCC 393) or *B. subtilis* (ATCC 23857), were activated twice in Tryptic Soy Broth at 37 °C during the log phase (18 hours). The bacterial suspensions were harvested by centrifuging at 13,000 x*g* for 10 minutes at 4 °C (Sorvall RC5C, USA), and the resulting supernatants were discarded. The pellets were washed twice with a distilled water solution and re-suspended in 1 mL of distilled water. Then, the ground hemp seeds (50 g seeds in 25 mL distilled water) were inoculated with 300 μL of the bacterial suspensions or co-cultures (7 log/mL of milk sample). The fermentation processes were carried out for 72 hours in a shaker incubator (New Brunswick Scientific, I24, Germany) at 37 °C and 150 rpm. After fermentation, the treated hemp seeds were dried at 45 °C in an oven and ground. The resulting fermented seeds were immediately used to produce fresh hemp milk samples.

**Production of GABA-enriched hemp milk**

The hemp seeds were ground in 5 times the volume of distilled water using a high-shear homogenizer (IKA, T20, Konigswinter, Germany) at a speed of 20,000 rpm for 5 minutes. The resulting mixture was then filtered through eight layers of cheesecloth to eliminate solid particles. The obtained crude milk-treated sonication (Sonics, VCX750, Newtown, USA) was in an ice bath at a 40% amplitude for 5 minutes. Then, raw hemp milk samples were heated at 90 °C for 10 min for pasteurization. The hemp milk samples derived from the solid-state fermented seeds by *L. casei* and *B. subtilis* and their co-cultures were denoted as FL, FB, and FBL, respectively. Additionally, the hemp milk samples from seeds germinating for 1, 2, and 3 days were labeled G1, G2, and G3, respectively. The hemp milk obtained from untreated seeds (U) was utilized as the control sample in the study.

**GABA content**

GABA content was assessed using the modified method of Ding et al. [3] Analysis was conducted using a high-performance liquid chromatography system (HPLC, Waters e2695) equipped with a ZORBAX Eclipse AAA reversed-phase column (3.5 μm), measuring 4.6 mm × 150 mm. The HPLC run time was under 30 minutes, with a column temperature maintained at 35 °C. To extract GABA, a 1 mL sample was mixed with 1 mL of 1 M NaHCO_3_ (pH 9) and 1 mL of dansyl chloride (1 mg/mL in acetone) in a graduated tube. The mixture was then subjected to a 10-minute reaction at 69 °C, followed by termination in an ice bath. For the chromatographic separation, two mobile phases were employed: 0.045 mol/L CH_3_COONa (pH 4) and acetonitrile at a flow rate of 0.5 mL/min. The GABA peak and its corresponding peak area were detected and quantified at 425 nm utilizing a Waters 2489 UV/Visible detector. The Limit of Quantification (*LOQ*) for DAD was 2.94 ppm. The external standard calibration curve was prepared between 5–250 ppm ranges.

**Zeta potential**

The Zeta potential of hemp milk samples was determined using a dynamic light scattering (Malvern, Zeta sizer NanoZS, UK) at a fixed scattering angle of 90° and with 658 nm wavelengths at 23 °C. The solution protein was characterized by absorption and refraction indexes of 0.001, 1.33, and 1.45, respectively.

**Droplet size**

The laser zeta sizer (Malvern, Zeta sizer NanoZS, UK) was utilized to measure the droplet size of hemp milk samples. Before analysis, hemp milk samples were diluted 100-fold using a sodium phosphate buffer (0.01 M pH 7.0). The formula (Eq. 1 and 2) was then employed to calculate the Sauter mean diameter ($D_{3,2}$) and weighted mean value by volume ($D_{4,3}$) values.

$D_{3,2}=\frac{\sum_{i} n_{i}{d_{i}^{3}}}{\sum_{i} n_{i}{d_{i}^{2}}}$ (1 )

$D_{4,3}=\frac{\sum_{i} n_{i}{d_{i}^{4}}}{\sum_{i} n_{i}{d_{i}^{3}}}$ (2)

where $d_{i}$ is the droplet diameter, $n_{i}$ is the number of droplets.

**Solid particle sedimentation**

Approximately 10 g of hemp milk samples were transferred to centrifuge tubes and centrifuged (Sorvall RC5C, USA) at 13,000 x*g* for 15 minutes. The weight of the supernatant was measured, and the solid precipitate at the bottom of the tubes was expressed as a percentage (% w/w) [4].

**ºBrix**

The ºBrix values representing the total soluble solids of milk samples were determined at room temperature (25ºC ± 1) using a refractometer (Atago, Japan) following the AOAC (2000).

**Free SH group content**

The determination of free SH group content was carried out using a modified version of [5]. Hemp milk samples were mixed in a buffer solution (pH 8.0; 0.086 M Tris, 0.09 M glycine, 4 mM EDTA) at a concentration of 0.25% w/v and stirred at room temperature for 1 hour. Subsequently, the resulting suspension was centrifuged (Sorvall RC5C, USA) at 13,000 x*g* for 25 minutes at 25 °C. To 3 mL of the supernatant, 0.03 mL of Ellman's reagent solution (4 mg DNTB/mL MeOH; 5,5-dithiobis (2–nitrobenzoic acid)) was added. After 15 minutes of reaction time, the absorbance of the sample was measured at 412 nm using a UV-spectrophotometer (Thermo, Spectronic Genesys 5, USA). A reagent blank (buffer solution) and a protein blank (Ellman's reagent) were employed to account for background absorbance. The protein content in the hemp milk samples was determined using the Bradford method. The range of free SH groups was expressed as µmol SH/gram protein, with a molar extinction coefficient of 13600 M^-1^cm^-1^, as calculated by Eq. 3.

Free SH (µmol/g protein) = Abs_412_ / (C x 13600) (3)

where Abs_412_ is the absorbance at 412 nm, and C is the protein concentration.

**Soluble protein content**

The method described by Morr et al. [6] was modified to assess the solubility of protein isolates. Precisely, hemp milk samples (10 mg/mL) were centrifugated at 13,000 xg, 25 °C for 15 minutes (Sorvall RC5C, USA), and the protein content in the supernatant was quantified using the Bradford method. Briefly, 150 µL of protein solution was mixed with 3 mL of ready-to-use Bradford reagent and incubated for 10 minutes. The protein content in the supernatant was determined at 595 nm using a spectrophotometer (Thermo, Spectronic Genesys 5, USA). The percentage of protein solubility was calculated by dividing the protein content in the supernatant by the total protein added to the dispersion, as shown in Eq. (4). Bovine serum albumin was used as a standard protein solution, and the correlation coefficient (R²) was found to be 0.9943.

$Protein solubility (\%) = \frac{Protein content in supernatant}{Initial protein content in sample} x 100$ (4)

**Peptide content**

The peptide content was determined according to the modified method of Liu et al. [7]. Hemp milk samples were mixed with a trichloroacetic acid (TCA) solution at a concentration of 0.4*M* to precipitate the proteins, and the resulting supernatant was collected through centrifugation (Sorvall RC5C, USA) at 13,000 x*g* for 10 minutes. The Bradford method was used to measure the collected supernatant.

**Total phenolic content**

The modified Folin-Ciocalteu standard method, as outlined by Kapoor and Feng [8], was utilized to determine the total phenolic content of hemp milk samples. The absorbance of the resulting mixtures was measured at 765 nm using a UV-spectrophotometer (Thermo, Spectronic Genesys 5, USA) and compared to a gallic acid standard curve. The total phenolic content was expressed as mg gallic acid per gram of dry-based samples.

**DPPH**

The measurement of protein scavenging activity on 1,1–diphenyl–2–picrylhydrazyl (DPPH) free radicals was conducted following the method of Brand-William et al. [9] with slight modifications. The absorbance of the solution was then recorded at 517 nm using a UV-spectrophotometer (Thermo, Spectronic Genesys 5, USA). For the control, methanol was added instead of the protein extract. The DPPH scavenging activity was expressed as a percentage using Eq. 6.

%DPPH scavenging activity= $\frac{Control-Sample}{\mathrm{Control}}x100$ (6)

where Control is the absorbance of the control; Sample is the absorbance of the protein.

**Statistical analysis**

All values were reported as mean ± standard deviation. Each sample was subjected to three replicates for both treatments and analyses. Statistical analysis was performed using R software (Version 3.6.3., R Foundation for Statistical Computing, Vienna, Austria). To determine the significance of differences between means, one-way ANOVA with Tukey's test was utilized with a 95% confidence interval.

**References**

[1] Miyahira RF, Lopes JDO, Antunes AEC (2021) The use of sprouts to improve the nutritional value of food products: A brief review. Plant Foods Hum Nutr 76:143–152. https://doi.org/10.1007/s11130-021-00888-6

[2] Xing Q, Dekker S, Kyriakopoulou K, Boom RM, Smid EJ, Schutyser MAI (2020) Enhanced nutritional value of chickpea protein concentrate by dry separation and solid-state fermentation. Innov Food Sci Emerg Technol 59:102269. https://doi.org/10.1016/j.ifset.2019.102269

[3] Ding J, Hou GG, Nemzer BV, Xiong S, Dubat A, Feng H (2018) Effects of controlled germination on selected physicochemical and functional properties of whole-wheat flour and enhanced γ-aminobutyric acid accumulation by ultrasonication. Food Chem. 243:214–221. https://doi.org/10.1016/j.foodchem.2017.09.128

[4] Valencia-Flores DC, Hernández-Herrero M, Guamis B, Ferragut V (2013) Comparing the effects of ultra-high-pressure homogenization and conventional thermal treatments on the microbiological, physical, and chemical quality of almond beverages, J Food Sci 78(2):199–205. https://doi.org/10.1111/1750-3841.12029

[5] Ellman GL (1959) Tissue sulfhydryl groups. Arch Biochem Biophys 82(1):70–77. https://doi.org/10.1016/0003-9861(59)90090-6

[6] Morr CV, German B, Kinsella JE, Regenstein JM, Buren JPV, Kilara A, Lewis BA, Mangino ME (1985) A collaborative study to develop a standardized food protein solubility procedure. J Food Sci 50:1715–1718. https://doi.org/10.1111/j.1365-2621.1985.tb10572.x

[7] Liu F, Chen Z, Shao J, Wang C, Zhan C (2017) Effect of fermentation on the peptide content, phenolics and antioxidant activity of defatted wheat germ. Food Biosci 20:141–148. https://doi.org/10.1016/j.fbio.2017.10.002

[8] Kapoor R, Feng H (2022) Characterization of physicochemical, packing and microstructural properties of beet, blueberry, carrot and cranberry powders: The effect of drying methods. Powder Technol 395:290–300. https://doi.org/10.1016/j.powtec.2021.09.058

[9] Brand-Williams W, Cuvelier ME, Berset C (1995) Use of a free radical method to evaluate antioxidant activity. LWT 28(1):25–30. https://doi.org/10.1016/S0023-6438(95)80008-5
