# Supplementary material for: γ-Aminobutyric Acid (GABA)-enriched Hemp Milk by Solid-state Co-fermentation and Germination Bioprocesses
Source: Plant Foods Hum Nutr. 2024 May 16;79(2):322–9. doi: 10.1007/s11130-024-01187-6 (PMC11178579; doi:10.1007/s11130-024-01187-6)
Supplement: Supplementary file 2 — Supplementary Material 2 [file 11130_2024_1187_MOESM2_ESM.docx]

**γ-Aminobutyric acid (GABA)-enriched hemp milk by solid-state co-fermentation and germination bioprocesses**

Gulsah Karabulut^a^, Boris V. Nemzer^b^, Hao Feng^c,^*

^a^Department of Food Engineering, Sakarya University, 54187, Sakarya / Turkey ^b^VDF FutureCeuticals, Inc., Momence, IL 60954, USA ^c^Department of Family and Consumer Sciences, North Carolina A&T State University, 27411, Greensboro, NC / USA

*Corresponding author: Hao Feng [hfeng@ncat.edu](mailto:hfeng@ncat.edu)

**Supplementary File 2**

**Acknowledgment**

The authors would also like to extend special thanks to the Crop Science Department (IL, USA) for providing the hemp seeds used in this study. Furthermore, the authors wish to acknowledge Dr. Miller from the Department of Food Science & Human Nutrition at the University of Illinois at Urbana-Champaign (IL, USA) for generously providing the bacterial cultures used in this research.

**Table 1S2.** GABA content of hemp seed and hemp milk samples.

| Hemp seed | GABA content (mg/100 g dry weight) | Hemp milk | GABA content  (mg/100 mL hemp milk) |
| --- | --- | --- | --- |
| U | 17.11 ± 0.98^a^ | U | <LOQ |
| G1 | 26.28 ± 3.40^b^ | G1 | 14.08 ± 1.16^a^ |
| G2 | 79.93 ± 6.21^c^ | G2 | 62.39 ± 4.71^b^ |
| G3 | 113.20 ± 3.61^d^ | G3 | 79.84 ± 0.89^b^ |
| FL | 128.51 ± 4.76^e^ | FL | 77.67 ± 3.60^c^ |
| FB | 152.38 ± 3.90^f^ | FB | 90.81 ± 0.87^d^ |
| FBL | 175.98 ± 9.16^f^ | FBL | 102.45 ± 1.33^e^ |

* LOQ for DAD was 2.94 ppm.

** U: untreated; G1, G2, and G3: germinated for 1 day, 2 days, and 3 days; FL, FB, and FBL: solid state fermented by *L. casei*, *B. subtilis,* and their co-cultures, respectively.

**Values are means ± SD. Letters indicate significant (*p* < 0.05) differences within the same column.
